# Decay of velvet worms (Onychophora), and bias in the fossil record of lobopodians

**DOI:** 10.1186/s12862-014-0222-z

**Published:** 2014-11-29

**Authors:** Duncan JE Murdock, Sarah E Gabbott, Georg Mayer, Mark A Purnell

**Affiliations:** Department of Geology, University of Leicester, University Road, Leicester, LE1 7RH UK; Animal Evolution and Development, Institute of Biology, University of Leipzig, Talstraße 33, D-04103 Leipzig, Germany

**Keywords:** Onychophora, Lobopodia, Fossil, Decay, Taphonomy, Exceptional preservation

## Abstract

**Background:**

Fossil lobopodians, including animals proposed to have close affinity to modern onychophorans, are crucial to understanding the evolution of the panarthropod body plan and the phylum-level relationships between the ecdysozoan groups. Unfortunately, the key features of their anatomy are un-mineralized and subject to biases introduced during death, decay and preservation, yet the extent to which these fossils have been affected by the processes of *post-mortem* decay is entirely untested. Recent experimental work on chordates has highlighted a profound bias caused by decay, resulting in the erroneous interpretation of badly decayed specimens as primitive members of a clade (stemward slippage). The degree to which this bias affects organisms other than chordates is unknown.

**Results:**

Here we use experimental decay of velvet worms (Onychophora) to examine the importance of decay bias in fossil lobopodians. Although we find stemward slippage is not significant in the interpretation of non-mineralized lobopodian fossils, the affect of decay is far from unbiased. Quantitative analysis reveals significant changes in body proportions during decay, a spectrum of decay resistance across anatomical features, and correlated decay of topologically associated characters.

**Conclusions:**

These results have significant implications for the interpretation of fossil lobopodian remains, demonstrating that features such as body outline and relative proportions are unreliable for taxonomy or phylogenetic reconstruction, unless decay is taken into account. Similarly, the non-independent loss of characters, due to juxtaposition in the body, during decay has the potential to bias phylogenetic analyses of non-biomineralized fossils. Our results are difficult to reconcile with interpretations of highly decay-prone tissues and structures, such as neural tissue, and complex musculature, in recently described Cambrian lobopodians. More broadly, we hypothesize that stemward slippage is unlikely to be a significant factor among the taphonomic biases that have affected organisms where decay-resistant features of the anatomy are rich in phylogenetically informative characters. Conversely, organisms which possess decay-resistant body parts but have informative characters concentrated in decay-prone tissues will be just as liable to bias as those that lack decay-resistant body parts. Further experimental analysis of decay is required to test these hypotheses.

**Electronic supplementary material:**

The online version of this article (doi:10.1186/s12862-014-0222-z) contains supplementary material, which is available to authorized users.

## Background

Fossilization of remains of non-biomineralized tissue is exceptional, but of critical importance. Such exceptionally preserved fossils from the Early Palaeozoic have transformed our understanding of the early evolution of many animal groups, providing constraints on analyses of evolutionary rates, and direct evidence of how the distinctive body plans of extant organisms evolved (e.g. arthropods [1]). Because they preserve body parts that would be expected to completely decompose soon after death, it is tempting to view these fossils as a faithful record of the anatomy of ancient animals and the diversity of faunas, but this is not something that can be assumed. Our view is obscured by the taphonomic filters of decay and fossilization, and it is vital that these filters are understood if we are to obtain a meaningful biological signal from these fossils.

Experimental taphonomy aims to tease apart these filters, with much effort focused on recognizing the biases introduced by decay [2,3]. Recent work has highlighted the importance of understanding the sequence of loss of anatomical characters [4,5], revealing a pattern of early decay of synapomorphies relative to symplesiomorphies that causes ‘stemward slippage’, whereby fossil taxa are placed in more basal positions than they should be due to non-random decay of the phylogenetic signal encoded in their anatomy. The pervasiveness of this bias, and whether some groups of animals are more susceptible than others is an area worth investigating; it may well be a very widespread phenomenon [6] and recent *in silico* simulated fossilization studies support this view [7].

Fossil lobopodians include animals proposed to have close affinity to modern onychophorans. They have a crucial role in understanding the evolution of the panarthropod body plan and the phylum-level relationships between the ecdysozoan groups [8], yet the extent to which these fossils have been affected by the processes of *post-mortem* decay is entirely untested. Here we investigate this through analysis of decay on onychophorans. The precise relationships between the fossil taxa occupying the onychophoran stem is under much debate [9–11], but modern onychophorans possess a range of characters including some that are unique to extant members of the clade and others that are shared by more inclusive groups. As such they are an appropriate model to investigate decay in both total-group onychophorans and their immediate sister groups. Our study is the first experimental analysis of stemward slippage in invertebrates, investigating character decay in onychophorans and the implications for understanding the fossil record of lobopodians.

## Results and discussion

**Figure 1:**
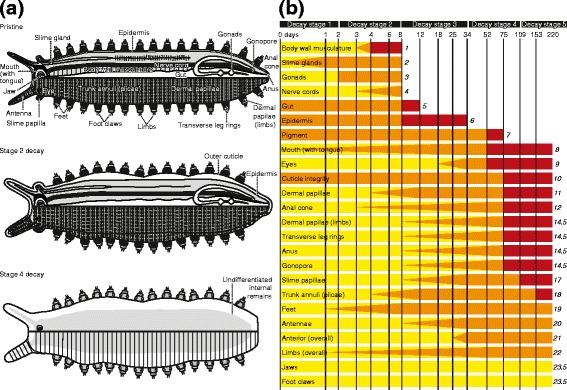
**Anatomy and decay of Onychophora. (a)** Idealized external (lower half) and internal anatomy (upper half) of Onychophora based on that of *E. rowelli*, with key anatomical characters labelled. Shown immediately *post-mortem* (upper), at the end of decay stage 2 (centre) and at the end of decay stage four (lower), to illustrate character loss and morphological deformation. **(b)** Decay sequence through time. Yellow = ‘pristine’; orange = ‘decaying’; red = some ‘lost’; bars terminate at first sampling interval where all are lost. Characters were initially ranked based on the timing of complete loss. Where this resulted in tied ranks, ties were broken based on the time of onset of loss. Remaining ties were broken based on the time at which all samples of a character exhibit decay, then the time of onset of decay. Rankings quoted at the termination of each bar.

**Figure 2:**
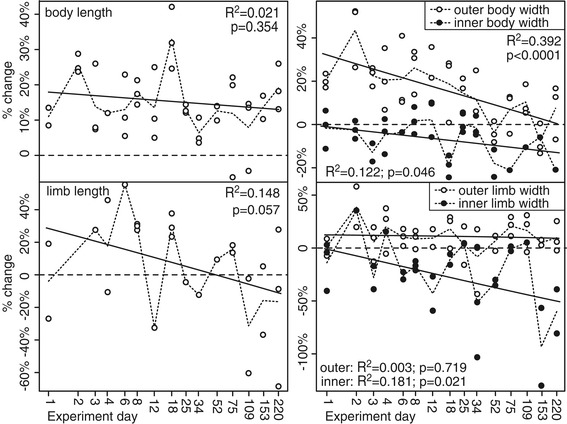
**Changes in overall morphology of** ***E. rowelli*** **during decay.** Change (%) is calculated as original length minus decayed length, divided by original length. Dotted lines join mean values; solid lines show linear regressions. **(a)** Change in the body length; R^2^ = 0.021, F = 0.881, df = 1,39, *p* = 0.354. **(b)** Change in body width; measurements were taken between the 7^th^ and 8^th^ limb pair, across the outer cuticle (open circles) and detached epidermis (solid); outer R^2^ = 0.392, F = 27.777, df = 1,43, *p* <0.0001; inner R^2^ = 0.122, F = 4.304, df = 1,31, *p* = 0.046. **(c)** Change in limb length (seventh pair); measurements were taken from base of the terminal pad to the junction between limb and trunk; R^2^ = 0.148, F = 4.003, df = 1,23, *p* = 0.057. **(d)** Change in the limb width (seventh pair); measurements were taken at the widest point of the limb, across the outer cuticle (open circles) and detached epidermis (solid); outer R^2^ = 0.003, F =0.131, df = 1,40, *p* = 0.719; inner R^2^ = 0.181, F = 5.966, df = 1,27, *p* = 0.021. F tests are for significance of slope of regression.

**Figure 3:**
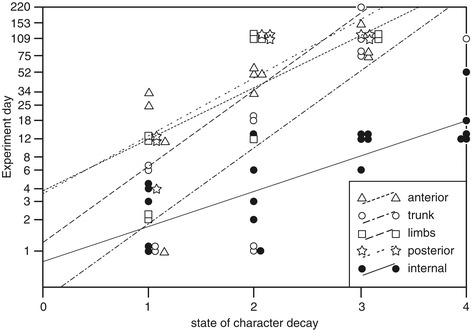
**Decay trajectory of characters grouped by body region.** States of character decay coded as: 0 = pristine, 1 = onset of decay, 2 = complete decay, 3 = onset of loss, 4 = complete loss. Jaws and terminal claws are not shown, as they are pristine throughout the experiment. Lines are linear regressions.

Within hours of death the onychophoran body flexes, generally a relative lengthening of the ventral side with arching back of the anterior and/or posterior resulting in an “S”-shaped, “U”-shaped or entirely curled body shape. (Onychophoran decay is summarized in Table 1 and Figures 1, 2 and 3. Details of the decay trajectory of each character examined are provided in Additional files 3, 4, 5 and 6). Flexing is most pronounced in the first 24 hours and continues to day 3, after which time the curvature of the body does not increase. The first signs of decay are the breakdown of the procuticle and separation of the outer cuticle and the epidermis. Body proportions also change (Figure 2 and Additional file 10): the trunk elongates, typically by 10 – 30%, and bloating results in an increase in width at the mid-point of the trunk to a similar degree; the limbs increase in length and width, typically by 10 – 25%. For inner and outer body width, and inner limb width, changes through time are reasonably approximated by a linear model, with significant decay slopes indicating that proportions vary with stage of decay (Figure 2; F-test results). For the body, this indicates initial expansion in outer width, followed by a decline towards original width, accompanied by decrease in the width of the inner body such that in all stages of decay there is a similar separation between epidermis and the outer cuticle. For the limbs, the outer cuticle is expanded slightly, but shows no evidence of a trend through time. The inner width significantly decreases, meaning that as decay proceeds, the gap between the epidermis and outer cuticle increases. A trend of initial increase in limb length followed by decrease falls just short of being statistically significant.

**Table 1:** **Decay stages for the onychophoran** ***Euperipatoides rowelli***

Stage 1	Days 0–2	Onset of breakdown of cuticle and pigment granules, soapy gut, loss of slime gland endpieces
Stage 2	Days 2–8	Onset of decay of remaining internal organs, advanced breakdown of cuticle and associated effects on limbs, mouth and trunk
Stage 3	Days 8–34	Total loss of distinct internal organs, onset of decay of all external characters (except jaws, claws and eyes)
Stage 4	Days 34–109	Total loss of epidermis, eyes become less distinct or lost, onset of pigment loss, head consistently decaying
Stage 5	Days 109–220+	Onset of loss of external ‘structural’ characters, complete loss of pigment granules, initial rupturing of outer cuticle

The experimental organisms did not decay completely during the sampled interval. However, based on previous experiments with polychaete worms [2] we predict that eventually only chitinous jaws and claws would remain.

The general trajectory of decay can be summarized in terms of three phases: (i) onset of decay of internal characters (decay stages 1 and 2, days 0 – 8), (ii) sharp increase in the proportion of decayed characters associated with the loss of internal organs (decay stage 3, days 8 – 34), and (iii) the progressive decay and subsequent loss of the remaining characters (decay stages 4 – 6, from day 34 onwards). These findings are supported by scanning electron microscope observations of fixed and dried specimens (Additional file 2).

In terms of stemward slippage, initial analysis yielded mixed results. Half the tests (those that favour non-homology of controversial characters) failed to reject the null hypothesis that character decay is random with respect to synapomorphic rank, but in contrast to previous studies, on chordates [4,5], correlations are negative (*r*_*s*_ = -0.488 – -0.482, p =0.017 – 0.019), i.e. the decay-prone characters tend to be ecdysozoan symplesiomorphies (largely internal organs), whilst most onychophoran apomorphies are relatively decay-resistant (e.g. jaws, foot claws, slime papillae). The remaining four tests (those that favour homology of controversial characters) return no significant correlation (Additional files 7 and 9).

These tests, however, could be considered as biased by the inclusion of onychophoran apomorphies and ecdysozoan symplesiomorphies — characters that are uninformative with regard to the placement of fossil lobopodians within panarthropods. When these are removed we are unable to reject the null hypothesis that character decay in onychophorans is random with respect to synapomorphic rank.

This result does not mean that decay is completely random. Decay of individual organs does not proceed in isolation, and although the onset of decay is not coincident, by day 12 all specimens sampled showed complete loss of all internal organs, and the onset of decay of the epidermis (total loss of epidermis occurred at day 52). After day 12, the body cavity was filled with a white soapy substance, which disaggregated during dissection; identification of individual organs was impossible. The substance filling the body cavity (after day 12) was very similar in appearance to the gut contents prior to day 12, suggesting that rupture of the gut wall and escape of gut contents is responsible for loss of definition of the internal anatomy. Figure 3 plots the time taken for each character to decay. Most exhibit approximately linear decay trajectories against the log sampling scale, but there are significant differences in slopes (i.e. rate of decay; F =2.65, df =4, p =0.042), with the internal organs decaying faster and following a separate trajectory to all other characters. This is supported by results of ANOVA; the time at which characters exhibit onset of loss is significantly different for different body regions (F = - 52.0, df = 4,13, p <0.0001; Tukey HSD indicates internal characters differ from all others; log data). Figure 3 shows that the trajectories of ‘trunk’ and ‘anterior’ characters are similar to each other, which may be due to the propensity for decay of all these characters being linked to the integrity of the cuticle. The posterior characters follow a similar trajectory to the characters associated with the limbs.

Tests for osmotic effects on *post-mortem* anatomical change show no significant differences between the progress of experiments performed in Robson’s saline prepared to both half (~3.7 ‰) and double (~14.6 ‰) concentration with those performed in standard solution. Likewise, experiments to test the effect of moult stage on *post-mortem* cuticle expansion show no significant differences in decay trajectory. Testing for variation in decay between taxa revealed that although the overall rate of decay was slower for the peripatid *Principapillatus hitoyensis* (Additional file 1), the sequence of decay does not differ from the peripatopsid *Euperipatoides rowelli* (Spearman’s rank correlation of decay ranks; *r*_*s*_ = 0.792; p <0.001). Thus, our detailed observations from *E. rowelli* can be considered as being more broadly representative of Onychophora.

## Conclusions

Our analysis of onychophorans and lobopodians finds no support for a bias towards the early decay of synapomorphies, as seen in chordates [4,5] and simulated in other taxa [7]. We are unable to reject the null hypothesis that character decay in onychophorans is random with respect to synapomorphic rank, and thus, with respect to the characters they share with onychophorans, stemward slippage is unlikely to be a significant factor among the taphonomic biases that have affected non-biomineralized lobopodian fossils. This largely reflects the fact that onychophoran apomorphies are characters of the cuticle, which are relatively decay-resistant. It follows from this that a similar pattern will characterize character decay and loss in all organisms where decay-resistant features of the anatomy are rich in phylogenetically informative characters. This includes other cuticularized animal clades, and those with character-rich mineralized skeletons (N.B. organisms which possess decay-resistant body parts but have informative characters concentrated in decay-prone tissues will be just as liable to bias as those that lack decay-resistant body parts). Of course, for clades with a good fossil record determining whether early loss from fossils of decay-prone, phylogenetically informative characters has caused phylogenetic decay bias will be difficult because characters that are not preserved will not have been identified as being informative for analysis of relationships among the fossil members of the clade. However, for many groups of organisms, the earliest parts of their phylogenetic history predate the origin of decay-resistant tissues. Where this is the case, and precisely because their informative characters must therefore be decay prone, it is these critical periods of evolutionary history that will be the most liable to bias.

While stemward slippage is unlikely to have significantly biased the lobopodian record, our results indicate that other potential phylogenetic and anatomical biases must be taken into account. It is clear that characters are not independent of one another as they decay, and this is significant. It has long been known that correlations between characters can cause problems for phylogenetic analysis (see ref [12] for discussion), leading to overweighting [13,14], less accurate tree topologies and tree lengths [15,16], and exaggerating support values by inflating the apparent number of synapomorphies at particular nodes. Previous discussion has focused on characters that are correlated for evolutionary or functional reasons, but correlated character loss through decay as documented here (i.e. resulting in non-independent absence from fossils) has similar potential to distort phylogenetic results: in much the same way that exclusion of fossils can lead to apparent morphological jumps in character acquisition (e.g. ref [17]), suites of characters that appear simultaneously in phylogenies because of their taphonomic association could distort perceived patterns of character evolution.

Considering the implications of our results for interpretations of lobopodian fossils, it is now possible to recognize a spectrum of relative decay resistance, providing additional data with which to test and constrain anatomical interpretations of fossil lobopodians: internal organs and tissues are the first to decay, followed by gut, epidermis, pigment, mouth and eyes, cuticle, and finally sclerotized jaws and claws (see Additional file 10 for further details). The majority of lobopodian fossils are known from Burgess Shale-type deposits, preserving compressed remains as organic carbon, authigenic clays, and iron minerals oxidized to pyrite [18]. Despite the exceptional quality of this preservation, no fossils are complete, and it is important for both anatomical and phylogenetic analyses to be able to distinguish characters that are missing because they rotted away from characters that were never present [19]. Our results confirm that some characters, such as jaws and claws, have high resistance to decay, indicating that their absence from exceptionally preserved fossils that preserve other decay-resistant characters can be taken to indicate genuine phylogenetic absence. That the fossil lobopodian *Antennacanthopodia* [20], for example, lacks jaws and claws, is most likely to be because they were never present.

Perhaps the most striking aspect of onychophoran decay is the breakdown of the procuticle, swelling of the outer cuticle and shrinking of the epidermis. This results in deformation of the external anatomy (bloating), with a corresponding collapse of the procuticle. This continues until the outer cuticle ruptures, after which gross anatomy becomes increasingly difficult to determine. Given that a great deal of cuticle structure is shared across the Ecdysozoa [21], this raises two potential problems with the interpretation of lobopodian fossils. Firstly, the preserved body outline may not be a faithful representation of the *in vivo* trunk and limb morphology, rather it will be relatively constricted at the points where limbs articulate with the trunk and inflated between limbs. Secondly, the fluid-filled cavity that results from the breakdown of the procuticle early in decay could be misinterpreted as the original body cavity. *Antennacanthopodia* from the Chengjiang biota again illustrates our point: we interpret the ‘body cavity’ and ‘body-wall musculature’ of *Antennacanthopodia* [20] as the result of separation and swelling of a decayed cuticle (the ‘body wall musculature’ and internal organs being the shrunken epidermis and its undifferentiated contents, the ‘body cavity’ being the space between the epidermis and the somewhat swollen outer cuticle). The same may be true of features in other lobopodians, including dark stains or ‘central canals’ within the limbs of, for example, *Hallucigenia* [22], *Microdictyon* [23] and *Onychodictyon* [24]. These stains are variously interpreted as alimentary diverticula, blood vessels, parts of the hydrostatic or nervous systems or leg musculature [8,25–27]. Our results suggest that before they are interpreted to have anatomical significance, the hypothesis that these stains represent nothing more than decayed and shrunken limbs within an outer cuticle must be rejected.

Changes in limb proportions are also pertinent to the hypothesis that fossil lobopodians represent two distinct groups. Long-limbed forms have lobopods that exceed the body diameter; they include *Hallucigenia*, *Paucipodia* and *Orstenotubulus* [28] from the Cambrian, and the recently described Carboniferous species *Carbotubulus waloszeki* [29]. Short-limbed forms have stubby, more conical lobopods, and range from the Cambrian through to the present day, including extant onychophorans. The relative proportions and disposition of limbs relative to the body (whether they are directed laterally or ventrally) have been used to construct scenarios of the evolution of arthropod locomotion (see e.g. ref [28]). Clearly, our results show that limb and body proportions can be altered by the process of decay; this cannot be explained by flexibility of limbs *in vivo* as deformation was observed in individuals *post-mortem* and exhibits a linear trend as decay progresses. Distortion can be reliably attributed to the decay process, and these changes in limb proportions must be borne in mind when interpreting morphotypes and potential modes of locomotion.

The rate at which characters decay also has implications for the interpretation of organs in fossil onychophorans and lobopodians. In decay stage 3, the individual internal organs all become indistinguishable, suggesting that the preservation of organs in fossils beyond the earliest stages of decay is unlikely (in our experiments they may be lost to decay as early as 8 days after death). Our results suggest that the presence of internal organs in fossil lobopodians would require highly exceptional circumstances, and interpretation of these characteristics implies very rapid mineralization — to replicate, or at least stabilize, internal anatomy — and a high degree of fidelity of preservation. This has significant implications: where decay prone characters (such as muscles) are preserved by diagenetic mineralization this must have occurred very soon after death; where evidence of rapid authigenic mineralization is absent, interpretations of fossilized structures as decay prone internal anatomy require close scrutiny. This also has a bearing on the longitudinal median structures interpreted as gut traces in a range of lobopodian taxa (e.g. *Aysheaia* [30], *Luolishania* [9], *Cardiodictyon* [27], *Microdictyon* [23], *Hallucigenia* [31], *Onychodictyon* [24]). All of these structures, particularly the inconsistent location of the median structure and movement towards the concave body side in ventrally flexed specimens of *Paucipodia* (interpreted as evidence for poor development of mesentery [26]), are consistent with them being the decayed and collapsed remains of the entire viscera. Undifferentiated remains of internal organs may also explain dark stains and irregular body extensions such as those in *Antennacanthopodia* [20] (Figures 1 and 2) and *Hallucigenia* [31]. Our results support the hypothesis that these extensions are the results of extrusion of undifferentiated but viscous body contents following the rupture of the cuticle in later stages of decay.

Similar caveats apply to interpretations of internal features such as preserved nervous system characters or muscles in fossil lobopodians. These decay very quickly after death, and this raises interesting questions concerning recent interpretations of nervous tissue in Cambrian arthropods, preserved as iron-rich minerals [26,32,33] or carbon film [34]. We must be careful not to overextend our results, but if nervous systems in all panarthropods exhibit similar propensity for decay as those of onychophorans, preservation by iron minerals or carbon-rich films of structures interpreted as nervous systems and muscles in Chengjiang arthropods is problematic and puzzling because these modes of preservation generally do not capture the most decay prone tissues. This implies four possible scenarios: i) nervous system characters in arthropods are more decay resistant than those of onychophorans; ii) preservation by iron minerals and carbon films in the Chengjiang occurred earlier, relative to decay, than in other Lagerstätten; iii) the nervous system tissues were preferentially preserved soon after death by an unknown mechanism and were subsequently replaced by iron minerals or carbon films; iv) the interpretations of nervous systems and muscles are incorrect. All of these hypotheses are tenable; further work is required to determine which of them is correct.

A suite of characters show an intermediate decay resistance, and although decay and preservation may conspire to preserve them, interpretation of their presence in fossils requires caution. For example, the shift in position of the mouth (terminal to ventral) is an apomorphy of various derived panarthropod clades [35,36]. However, the position of the mouth in moderately decayed onychophorans cannot be differentiated, and it is unlikely to be a reliable character in lobopodians unless features that are equally or more decay-prone are present (i.e. the mode of preservation of the deposit facilitates preservation of decay-prone characters). At a similar point in the decay of onychophorans, eyes are lost. This contrasts with work showing that eyes in arthropods are resistant to decay [3]. Onychophorans and arthropods share ocellus-like eyes (although arthropods also bear compound eyes) [37,38], but our results suggest caution should be used when making inferences regarding lobopodian ‘eyes’. By the same token, where ocellus-like eyes in a lobopodian fossil are unequivocally preserved, they can serve as evidence for genuine phylogenetic absence of characters known to be more decay resistant (e.g. antennae or slime papillae). Similar problems face interpretation of the distribution of colour in fossil lobopodians. The mobility of pigment granules within decaying onychophorans, prior to their ultimate degradation, may result in patterns of colour in fossils that are not representative of the original pigmentation or anatomical features.

It has been noted that the majority of soft-tissue preservation in animals in the Palaeozoic occurs in groups with a decay-resistant outer cuticle [39], such as arthropods, polychaetes and priapulids. Experimental analyses of soft-tissue decay have been carried out previously on the polychaete annelid *Nereis* [2] and the crustacean arthropods *Crangon* and *Palaemon* [3]. With the addition of the onychophoran data here a number of common patterns can be identified (see Additional file 10). Although differences in decay trajectory are evident between these three studies, due in part to the anatomical differences between each experimental organism, the pattern of early loss of internal anatomy and relative longevity of the cuticle seems robust. In addition, the topological decay bias, described above, demonstrates the importance of the cuticle in onychophoran decay. This supports the hypothesis [39] that a decay-resistant cuticle is integral to some pathways of exceptional preservation, and that the interpretation of internal anatomy in instances of soft-tissue preservation of cuticularized animals should proceed with caution.

Our results, and general considerations of decay resistance, suggest that some groups of animals are much more prone to stemward-slippage than others, but they also confirm that decay resistance of non-biomineralized characters is difficult to predict in the absence of empirical evidence. Further experimental analyses of decay are required to establish the prevalence of this phylogenetic bias across the tree of life.

## Methods

Our experiments focus on adult specimens of the peripatopsid *Euperipatoides rowelli* (54 individuals, ~24 months old). Accessory experiments were carried out on adult specimens of the peripatid *Principapillatus hitoyensis* (5 individuals). Although modern onychophorans are terrestrial, they provide the only extant analogues for many of the anatomical characters of extinct marine lobopodians. The experimental methodology generally followed Sansom *et al.* [4,5]. All animals used in experiments were euthanized by asphyxiation in nitrogen gas. Specimens were decayed in individual 58 × 38 × 22 mm polystyrene boxes with closely-fitting lids, filled with a physiological buffer solution that replicates the composition of onychophoran haemolymph (= Robson’s saline) [40], with the omission of glucose. This isotonic solution (~7.3 ‰) was used to limit the effect of osmosis during the experiment (see below). The intention of these experiments was to establish a null-model for the sequence of decay of characters, not to mimic the process of fossilization, and as such the minimum number of experimental variables was used. Therefore, no bacterial inocula or antifungal agents were added, no attempts were made to disrupt the endogenous bacteria of the specimen, no additional substrate or sediment was added that may have altered the chemical environment in which the specimens decayed. The box lids were sealed closed with silicon grease (Ambersil M494); oxygen saturation, irrespective of its initial value, converges rapidly upon anoxia [3]. Of course, for particular fossils the individual decay trajectories of animals decaying in specific depositional environments will vary; our experiments are designed to establishing the sequence of character loss without any other confounding variables. Without these data determining the role of decay in particular cases, and testing hypotheses that specific environmental conditions enhanced preservation, are totally intractable. Sampling intervals were spaced according to a logarithmic model, with high initial sampling frequency to capture early rapid decay [4,5]. At each sampling interval specimens were photographed prior to and after dissection, and the condition of external and internal anatomical features logged and described. Internal and external anatomy of onychophorans (Figure 1) was documented and morphological characters were categorized by position in the organism: anterior, trunk, limbs, posterior, and internal organs. For morphological decay, each character for each sample for each interval was scored according to three defined states: pristine (same condition as at death), decaying (morphology altered from that of condition at death) or lost (no longer observable or recognizable) (Additional files 3, 4 and 5). For statistical analysis, characters were arranged according to the sequence in which they decay and ranked from the most decay-prone to the most decay-resistant (Figure 1; Additional file 6). The decay rank and synapomorphic rank (Additional file 7) of characters were then compared using Spearman’s rank correlation, with synapomorphic rank calculated for each of four alternative hypotheses of panarthropod relationships. Where the homology of characters is contested, we conducted separate analyses treating them either as homologies or homoplasies (see Additional file 10). This yielded 8 correlation tests. Linear dimensions were measured from photographic images of each specimen (one captured immediately after death, one prior to dissection), with body length taken from the anteriormost (not including antennae) to posteriormost tip of the trunk, along the midline. Accessory experiments were carried out to test for osmotic effects on *post-mortem* anatomical change in onychophorans, to test the effect of moult stage on *post-mortem* cuticle expansion, and to test for variation in decay between taxa (see Additional file 10).

## Additional files

Additional file 1:
**Proportion of characters exhibiting decay through the experiment.** A comparison of data from the main experiment with the peripatopsid *Euperipatoides rowelli* (circles, solid) and the peripatid *Principapillatus hitoyensis* (triangles, dashed). For each specimen for each sample decaying characters were scored 0.5 and lost characters scored 1.0; the sum of these was divided by the number of observed characters to give the proportion of decay. Open shapes represent original data, lines represent mean values. Although the degree of decay is different, the general trajectories are the same for both sets of experiments. Additional file 2:
**Microstructural details of the anatomy of** ***Euperipatoides rowelli*** **throughout the experiment.** Scanning electron micrographs. A. Tip of the antenna with sensilla after 30 days. B. Inner surface of epidermis is smooth after 2 days. C. Breakdown of procuticle after 2 days. D. Fibrous texture of inner cuticle after 30 days. E. Outer cuticle after 75 days. F. Inner cuticle after 75 days. G. Jaws after 2 days. H. Foot claws after 30 days. Scale bar represents: 160 μm, A; 300 μm, B; 200 μm, C,F; 500 μm, D; 360 μm, E; 430 μm, G; 170 μm, H. Additional file 3:
**Graphical representation of the characterization of decay state for most decay-prone characters recorded in the experiments.** ‘Last pristine’: an example from the latest sample where the character was still deemed to be indistinguishable from that immediately after death. ‘First decaying’ and ‘Last decaying’: examples of the earliest and latest example where the character was deemed to have been altered from the condition immediately after death, but still present and recognizable. ‘Lost’: an example of the appearance of characters deemed to be no longer present or not sufficiently recognizable; location of absent characters inferred from topological relations and position in the carcass. Additional file 4:
**Graphical representation of the characterization of decay state for the characters of intermediate decay resistance recorded in the experiments.** Details as in Additional file 3. Additional file 5:
**Graphical representation of the characterization of decay state for most decay-resistant characters recorded in the experiments.** Details as in Additional file 3, grey boxes denote states not reached during the experiment. Additional file 6:
**Raw data collected during the experimental decay of** ***E. rowelli*** **.** Three specimens where sampled at each sampling interval, and the state of twenty-four characters was recorded. Yellow = ‘pristine’; orange = ‘decaying’; red = ‘lost’; green = unable to record or identify. Characters were initially ranked based on the timing of complete loss. Where this resulted in tied ranks, ties were broken based on the time of onset of loss. Remaining ties were broken based on the time at which all samples of a character exhibit decay, then the time of onset of decay. Rankings quoted at the termination of each bar. Decay resistance increases down the diagram. Additional file 7:
**Four alternative hypotheses of relationships within the Ecdysozoa, with characters from the decay experiments mapped onto the topologies.** In each case two alternatives are given, one favouring the interpretation of some characters as homoplastic (square brackets), i.e. independently derived in two or more groups. The other favouring an interpretation of some characters as homologous between several groups (curly brackets), and in some instances invoking secondary loss in one or more groups. Phylogenetic rank for each set of characters at a node is shown, and used in comparisons with decay rank. AC, anal cone; AS, anus; AT, antennae; CU, integrity of cuticle; DL, dermal papillae on limbs; DP, dermal papillae on trunk surface; EP, epidermis; EY, simple, ocellus-like eyes; FC, foot claws; FT, feet; GN, gonads (most experimental animals were males); GP, gonopore; GT, gut; JW, jaws; LO, overall morphology of limbs; LP, spinous pads on limbs; LR, transverse leg rings; MO, mouth with tongue; MU, body wall musculature; NC, ventral nerve cords (without segmental ganglia); PG, pigment granules; SG, slime glands; SP, slime papillae; TA, trunk annuli (plicae). Additional file 8:
**Table of character transformation and loss throughout decay experiments in the onychophoran** ***Euperipatoides rowelli*** **.**
Additional file 9:
**Table of phylogenetic distribution of characters among panarthropods and cycloneuralians.**
Additional file 10:
**Additional background, results and details of the methodology.**
