# Supplementary figures and images for: Decay of velvet worms (Onychophora), and bias in the fossil record of lobopodians
Source: BMC Evol Biol. 2014 Nov 29;14:222. doi: 10.1186/s12862-014-0222-z (PMC4266977; doi:10.1186/s12862-014-0222-z)

Proportion of decay through experiment

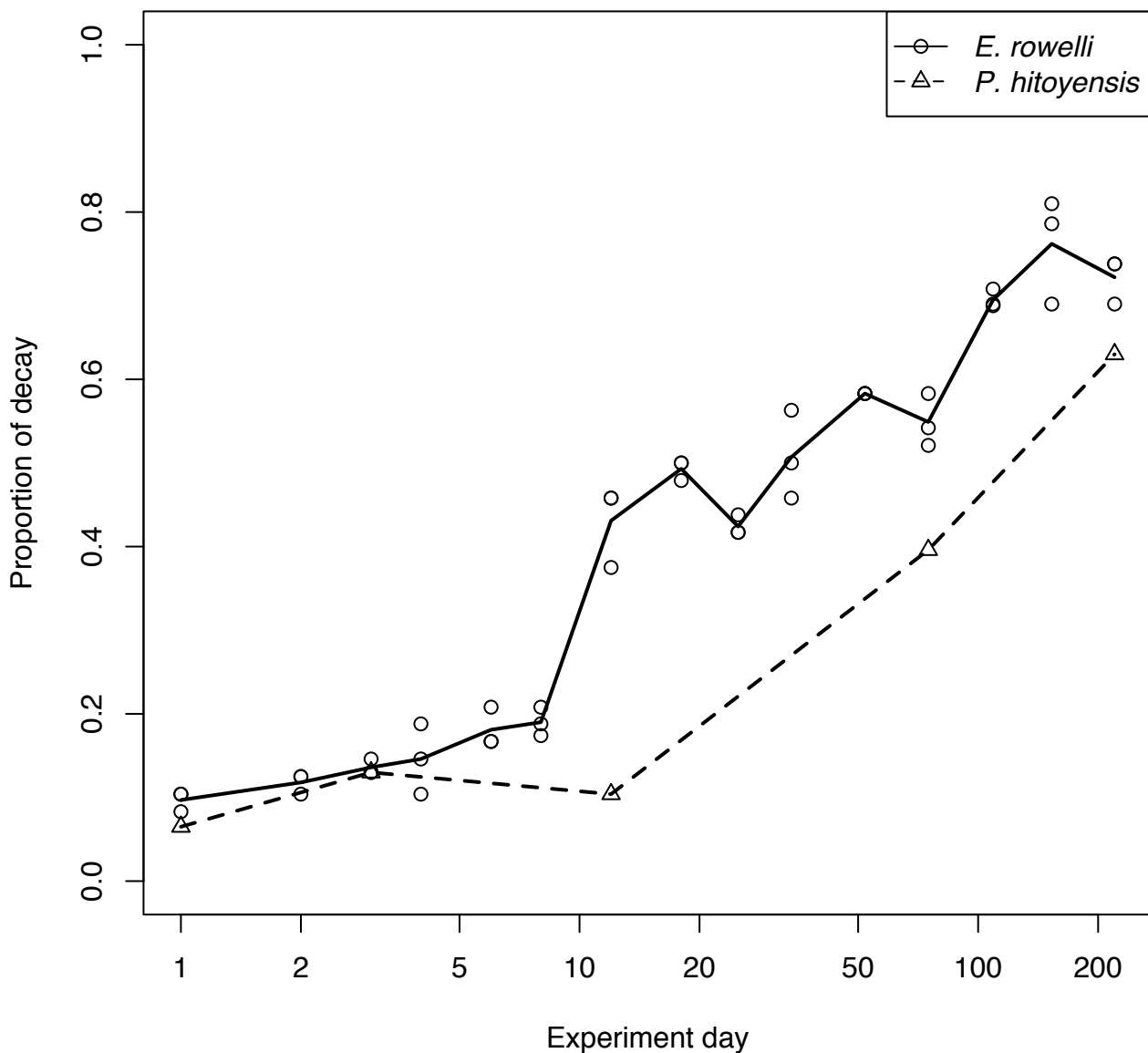

Supplement: Additional file 1: — Proportion of characters exhibiting decay through the experiment. A comparison of data from the main experiment with the peripatopsid Euperipatoides rowelli (circles, solid) and the peripatid Principapillatus hitoyensis (triangles, dashed). For each specimen for each sample decaying characters were scored 0.5 and lost characters scored 1.0; the sum of these was divided by the number of observed characters to give the proportion of decay. Open shapes represent original data, lines represent mean values. Although the degree of decay is different, the general trajectories are the same for both sets of experiments. [file 12862_2014_222_MOESM1_ESM.pdf]

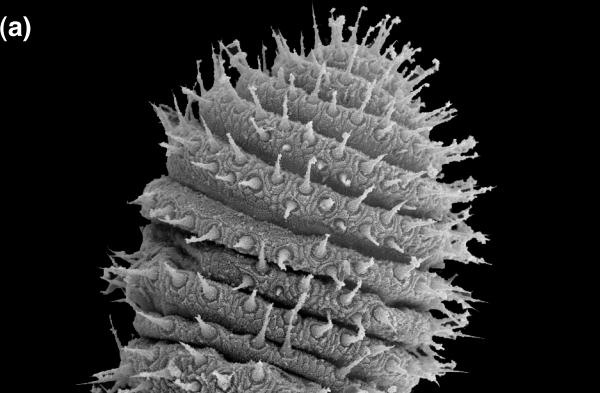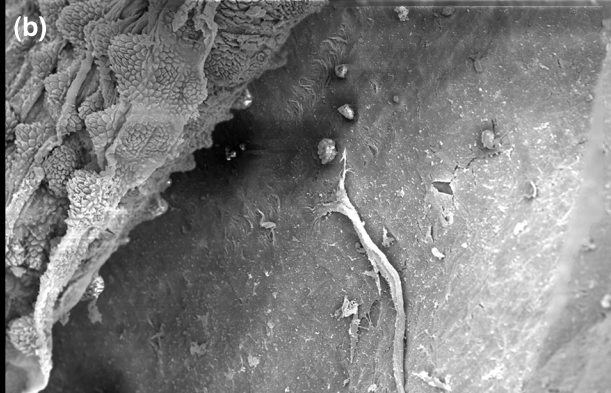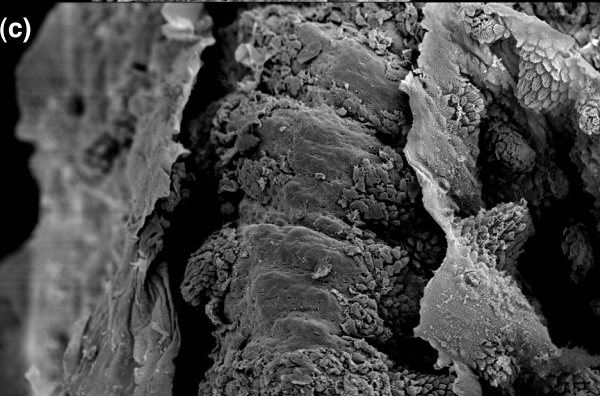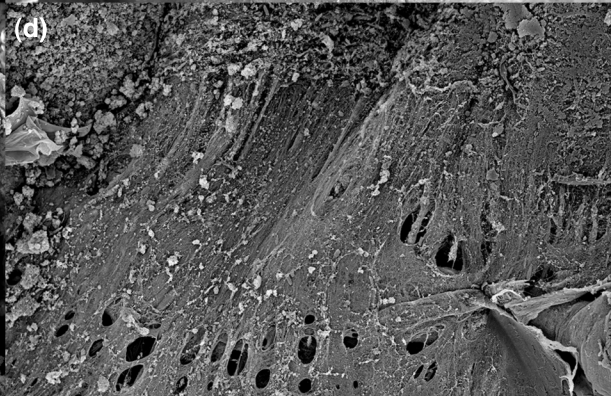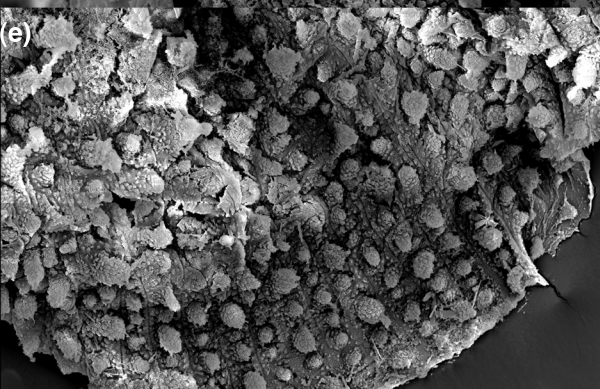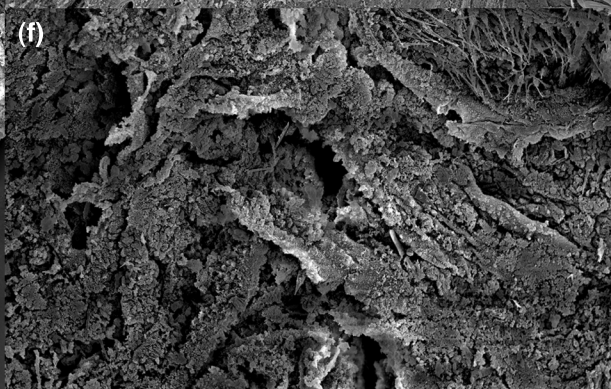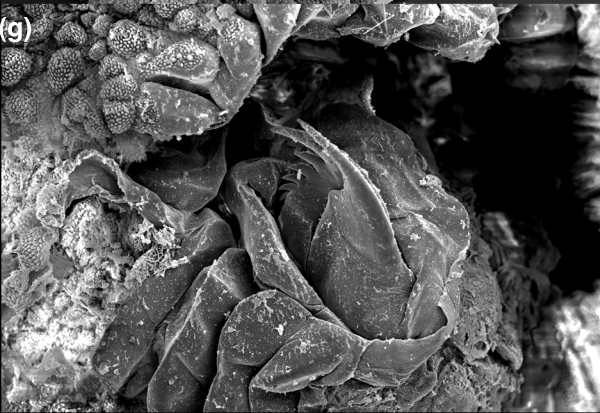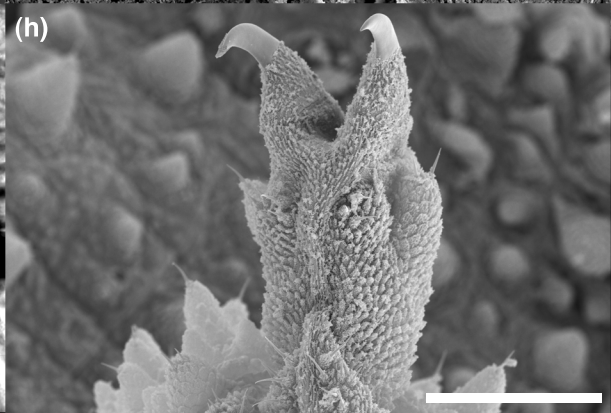

Supplement: Additional file 2: — Microstructural details of the anatomy of Euperipatoides rowelli throughout the experiment. Scanning electron micrographs. A. Tip of the antenna with sensilla after 30 days. B. Inner surface of epidermis is smooth after 2 days. C. Breakdown of procuticle after 2 days. D. Fibrous texture of inner cuticle after 30 days. E. Outer cuticle after 75 days. F. Inner cuticle after 75 days. G. Jaws after 2 days. H. Foot claws after 30 days. Scale bar represents: 160 μm, A; 300 μm, B; 200 μm, C,F; 500 μm, D; 360 μm, E; 430 μm, G; 170 μm, H. [file 12862_2014_222_MOESM2_ESM.pdf]

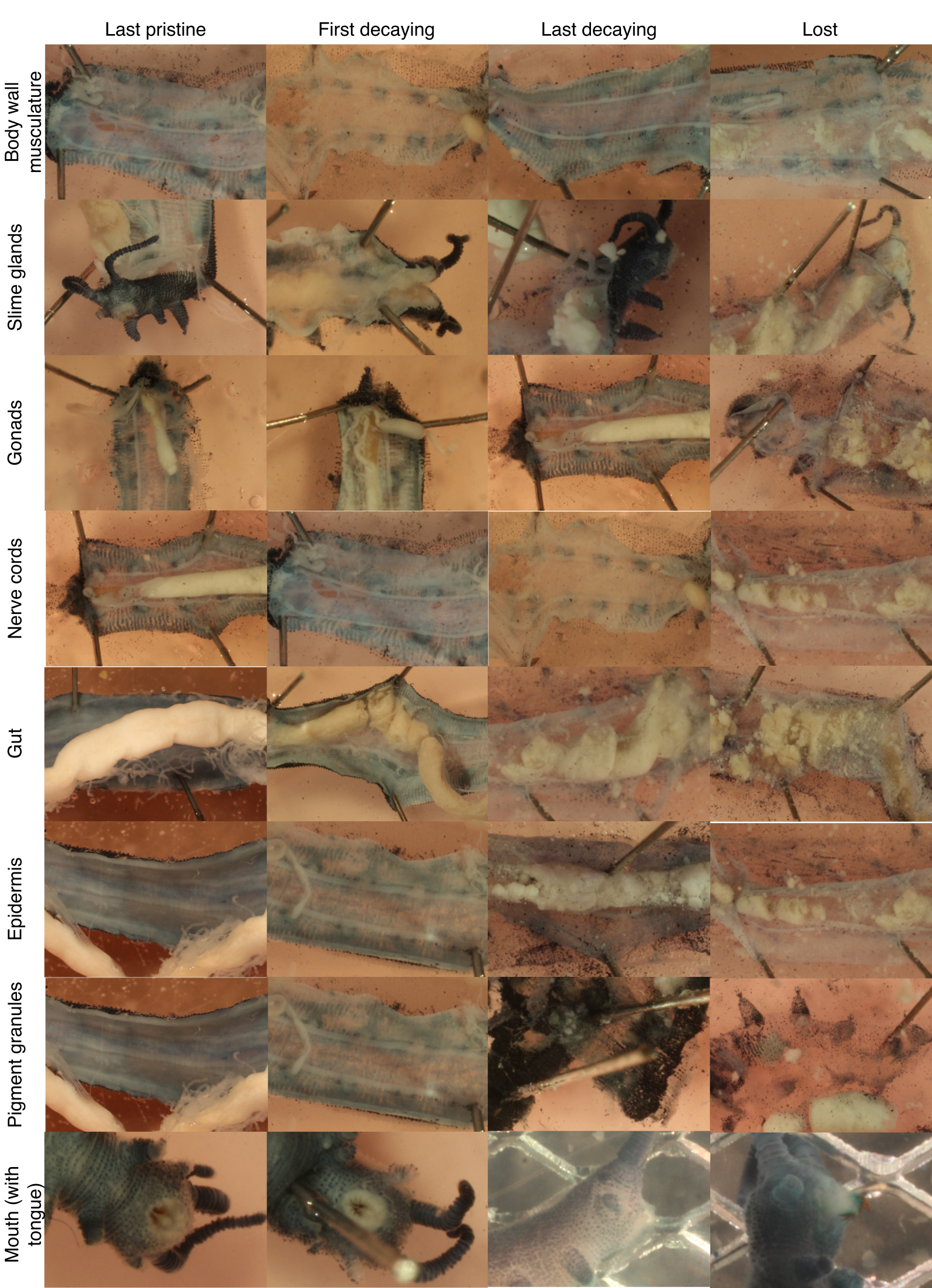

Supplement: Additional file 3: — Graphical representation of the characterization of decay state for most decay-prone characters recorded in the experiments. ‘Last pristine’: an example from the latest sample where the character was still deemed to be indistinguishable from that immediately after death. ‘First decaying’ and ‘Last decaying’: examples of the earliest and latest example where the character was deemed to have been altered from the condition immediately after death, but still present and recognizable. ‘Lost’: an example of the appearance of characters deemed to be no longer present or not sufficiently recognizable; location of absent characters inferred from topological relations and position in the carcass. [file 12862_2014_222_MOESM3_ESM.pdf]

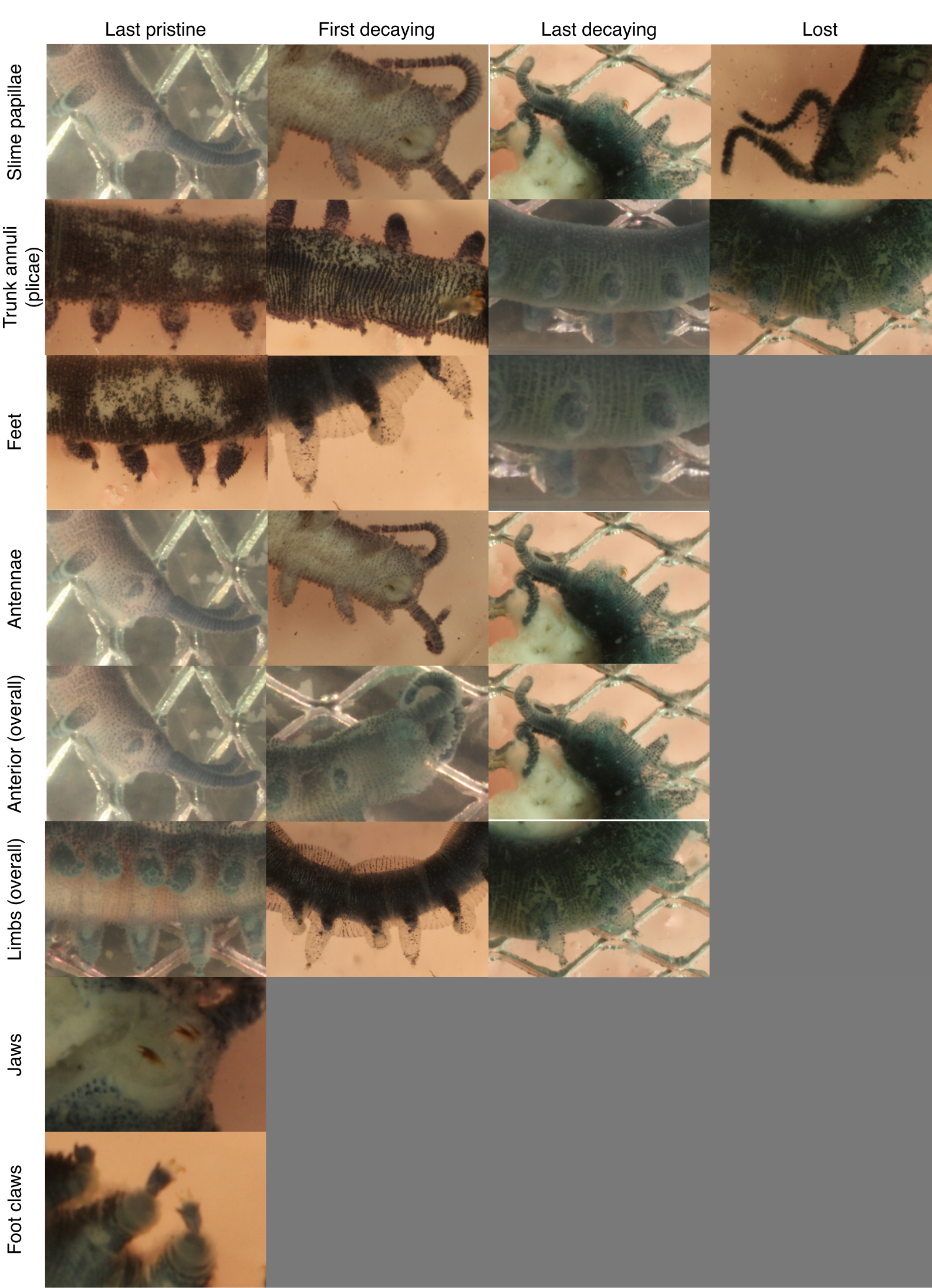

Supplement: Additional file 5: — Graphical representation of the characterization of decay state for most decay-resistant characters recorded in the experiments. Details as in Additional file 3, grey boxes denote states not reached during the experiment. [file 12862_2014_222_MOESM5_ESM.pdf]

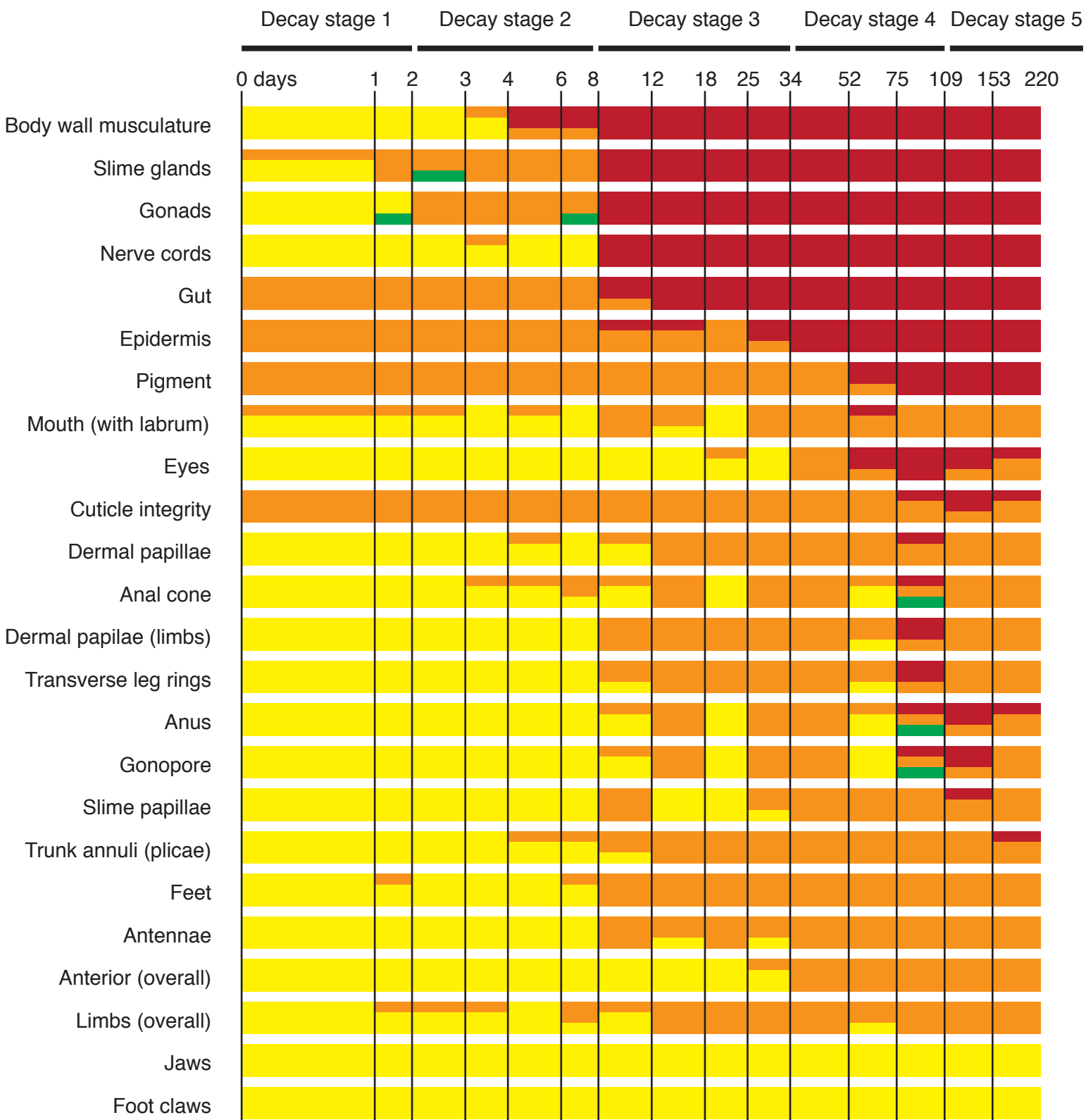

Supplement: Additional file 6: — Raw data collected during the experimental decay of E. rowelli . Three specimens where sampled at each sampling interval, and the state of twenty-four characters was recorded. Yellow = ‘pristine’; orange = ‘decaying’; red = ‘lost’; green = unable to record or identify. Characters were initially ranked based on the timing of complete loss. Where this resulted in tied ranks, ties were broken based on the time of onset of loss. Remaining ties were broken based on the time at which all samples of a character exhibit decay, then the time of onset of decay. Rankings quoted at the termination of each bar. Decay resistance increases down the diagram. [file 12862_2014_222_MOESM6_ESM.pdf]

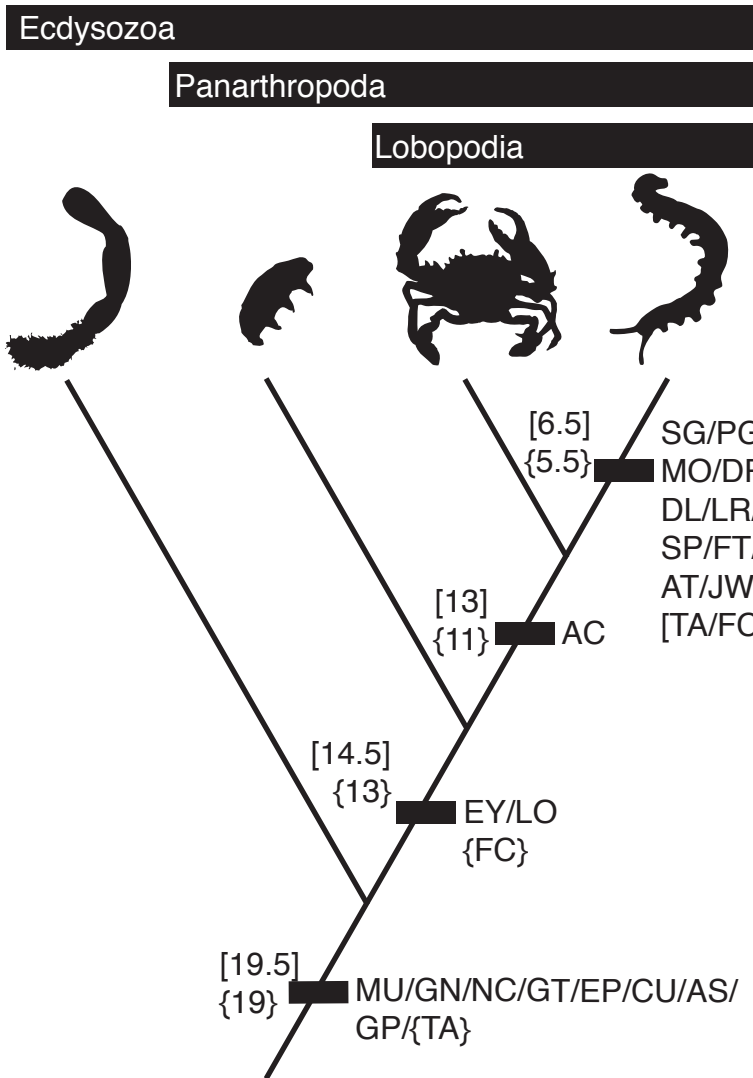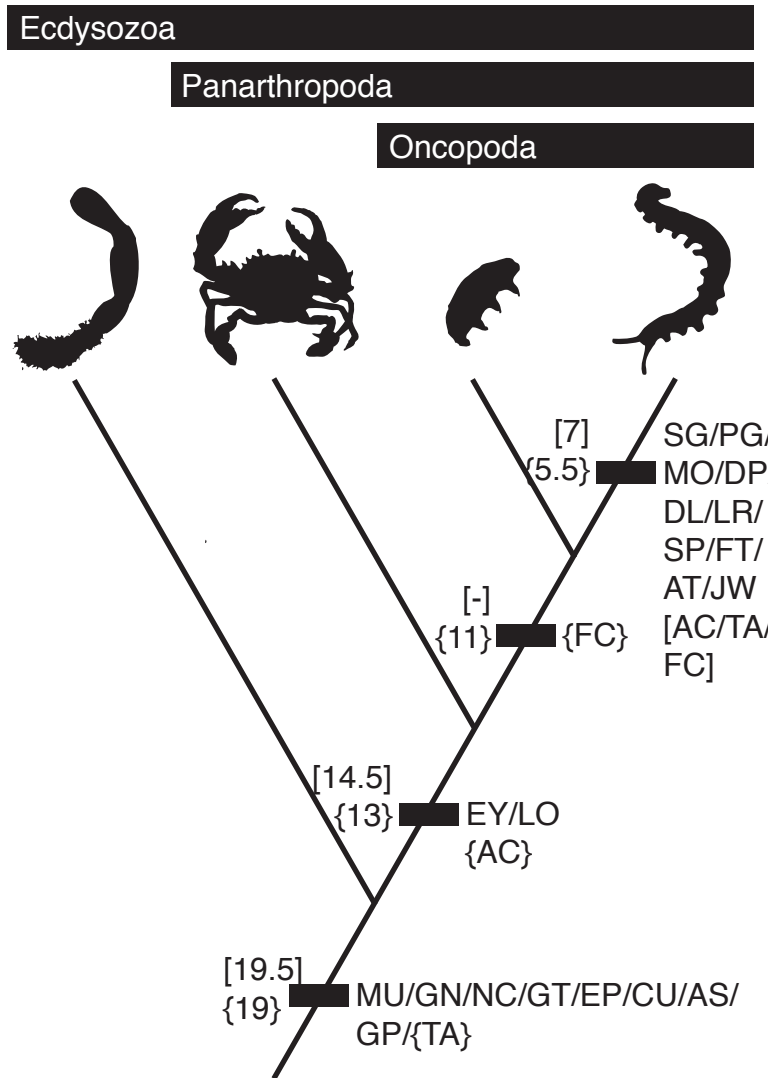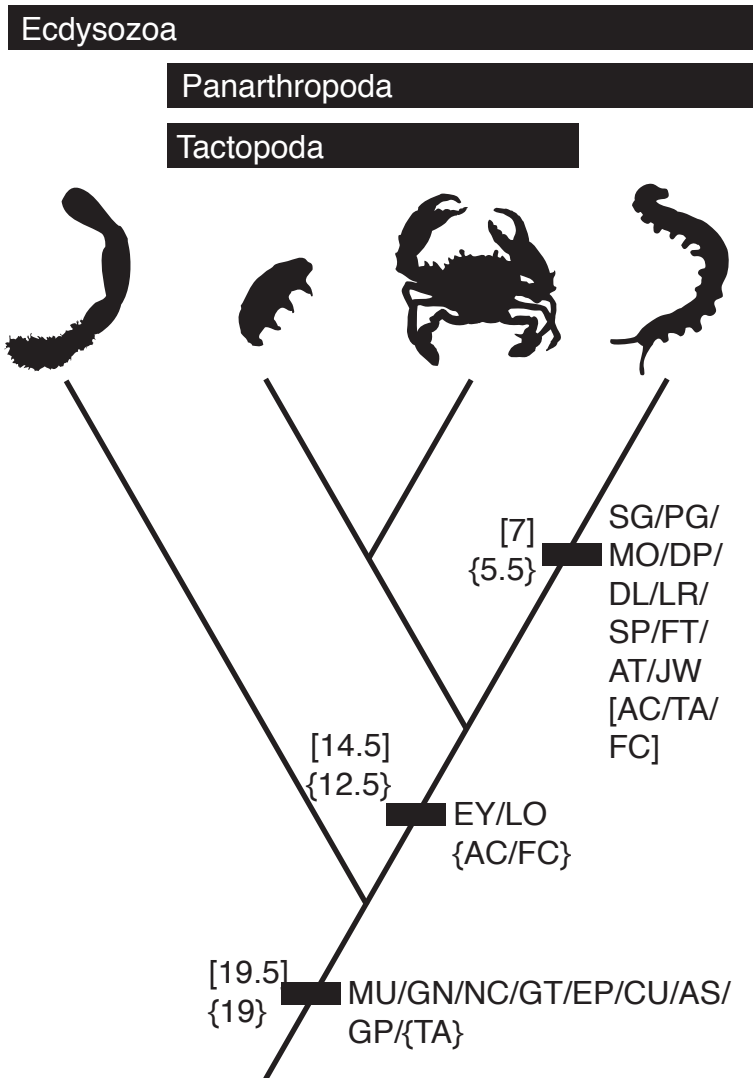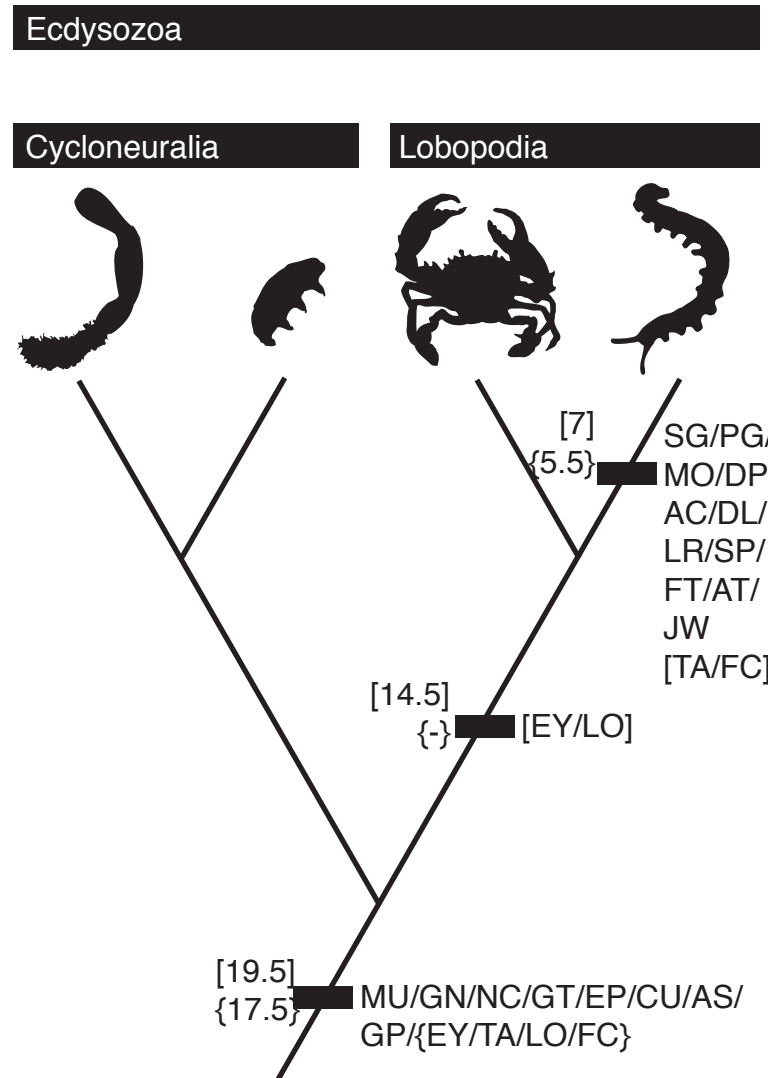

Supplement: Additional file 7: — Four alternative hypotheses of relationships within the Ecdysozoa, with characters from the decay experiments mapped onto the topologies. In each case two alternatives are given, one favouring the interpretation of some characters as homoplastic (square brackets), i.e. independently derived in two or more groups. The other favouring an interpretation of some characters as homologous between several groups (curly brackets), and in some instances invoking secondary loss in one or more groups. Phylogenetic rank for each set of characters at a node is shown, and used in comparisons with decay rank. AC, anal cone; AS, anus; AT, antennae; CU, integrity of cuticle; DL, dermal papillae on limbs; DP, dermal papillae on trunk surface; EP, epidermis; EY, simple, ocellus-like eyes; FC, foot claws; FT, feet; GN, gonads (most experimental animals were males); GP, gonopore; GT, gut; JW, jaws; LO, overall morphology of limbs; LP, spinous pads on limbs; LR, transverse leg rings; MO, mouth with tongue; MU, body wall musculature; NC, ventral nerve cords (without segmental ganglia); PG, pigment granules; SG, slime glands; SP, slime papillae; TA, trunk annuli (plicae). [file 12862_2014_222_MOESM7_ESM.pdf]
