# Supplementary material for: Decay of velvet worms (Onychophora), and bias in the fossil record of lobopodians
Source: BMC Evol Biol. 2014 Nov 29;14:222. doi: 10.1186/s12862-014-0222-z (PMC4266977; doi:10.1186/s12862-014-0222-z)
Supplement: Additional file 4: — Graphical representation of the characterization of decay state for the characters of intermediate decay resistance recorded in the experiments. Details as in Additional file 3. [file 12862_2014_222_MOESM4_ESM.pdf]

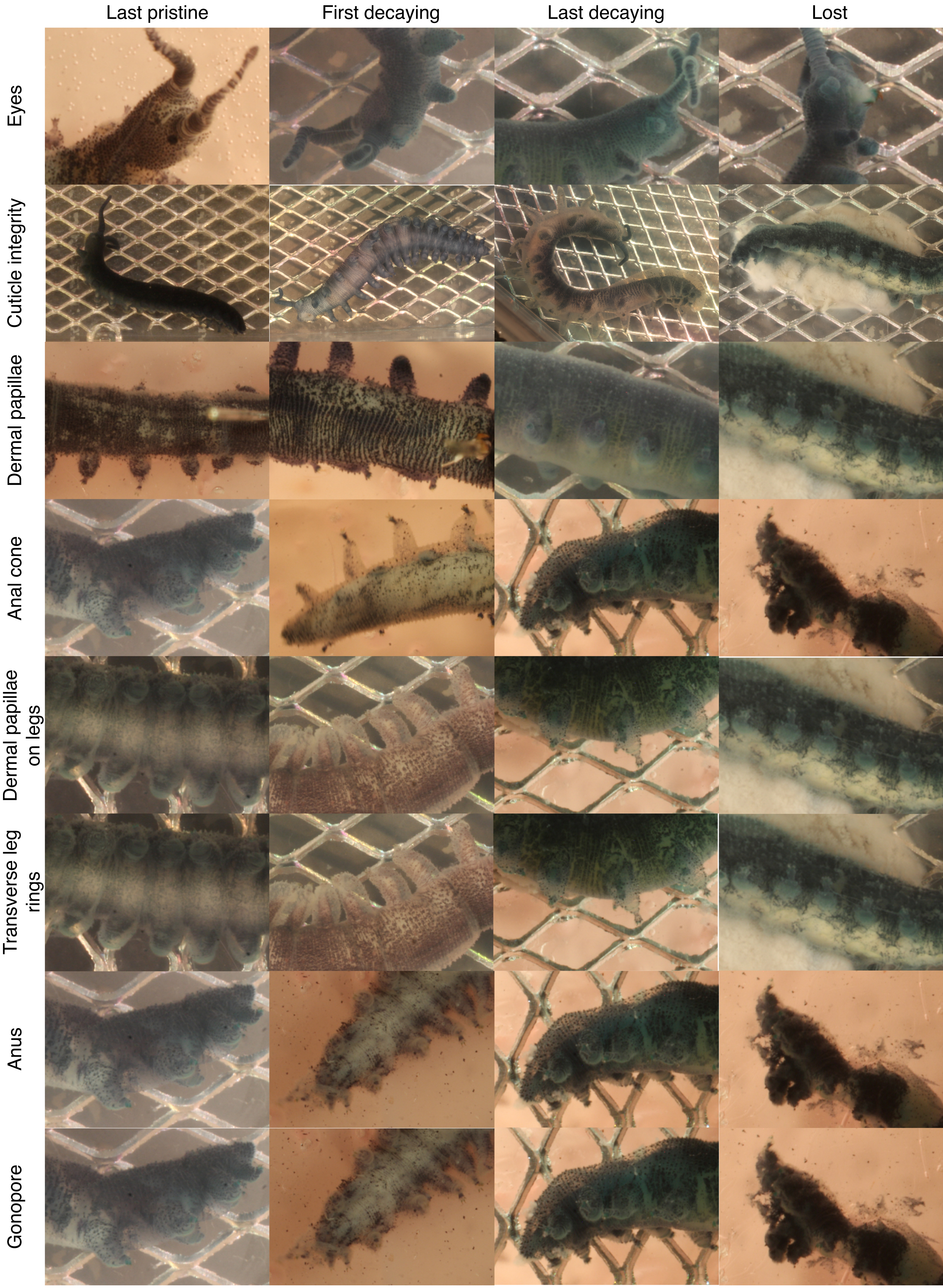
Last pristine
First decaying
Last decaying
Lost
Eyes
Cuticle integrity
Dermal papillae
Anal cone
Dermal papillae on legs
Transverse leg rings
Anus
Gonopore
