# Supplementary material for: Decay of velvet worms (Onychophora), and bias in the fossil record of lobopodians
Source: BMC Evol Biol. 2014 Nov 29;14:222. doi: 10.1186/s12862-014-0222-z (PMC4266977; doi:10.1186/s12862-014-0222-z)
Supplement: Additional file 8: — Table of character transformation and loss throughout decay experiments in the onychophoran Euperipatoides rowelli . [file 12862_2014_222_MOESM8_ESM.pdf]

Additional file 8: Character transformation and loss throughout decay experiments in the onychophoran *Euperipatoides rowelli*.

	**'Pristine' condition**	**'Decaying' condition**	**'Lost' condition**
Overall morphology			
Integrity of cuticle	All layers intact	Breakdown of procuticle and separation of outer cuticle and procuticle from epidermis	Rupture of outer cuticle, and fibrous texture
Anterior	Morphology faithfully replicated by all layers of cuticle (but allowing for some detachment of layers)	Morphology only visible in outer cuticle	Individual structures indistinct
Limbs	Morphology faithfully replicated by all layers of cuticle (but allowing for some detachment of layers)	Clear deformation of limb and complete detachment of foot base	Limbs indistinguishable
Posterior	Morphology faithfully replicated by all layers of cuticle (but allowing for some detachment of layers)	Morphology only visible in outer cuticle	Individual structures indistinct
Head structures			
Antennae	As at death, but allows for some whitening, and curling	Morphology only distinct in outer cuticle	No longer identifiable
Slime papillae	As at death, but allows for some whitening, and shape change	Morphology only distinct in outer cuticle	No longer identifiable
Jaws	As at death, but allows for relative movement of elements	Stage not reached during experiment	Stage not reached during experiment
Mouth (with	As at death but	Swelling of tongue	Only identifiable

tongue)	allows for whitening and detachment of surrounding cuticle		by the presence of jaws
Eyes	Clearly visible dark eye spots	Reduction in darkness of pigment and loss of distinct outline	No longer visible
Trunk structures			
Dermal papillae	As at death, but allows for whitening and some shape distortion associated with cuticle decay	Some lost or indistinct, significant swelling and distortion	Effacement of trunk
Pigment	Pigment contained within pigment granules, strong dark-blue coloration	Pigment granules disturbed or burst, lightening of dorsum, venter becomes pinker	Complete loss of blue coloration and break-up of distinct granules
Trunk annuli (plicae)	As at death, but allowing for kinking associated with whole body deformation	Annuli become wavy and/or indistinct	Effacement of trunk
Limbs			
Transverse leg rings	As at death, but allowing for kinking associated with whole limb deformation	Leg rings become wavy and/or indistinct	Effacement of limbs
Dermal papillae on limbs	As at death, but allows for whitening and some shape distortion associated with cuticle decay	Some lost or indistinct, significant swelling and distortion	Effacement of limbs
Foot claws	As at death, but allows for relative movement of elements	Stage not reached during experiment	Stage not reached during experiment
Feet	Morphology faithfully replicated by all layers of cuticle (but allowing for some detachment of	Morphology only visible in outer cuticle	No longer identifiable

	layers)		
Posterior body region			
Anus	Morphology faithfully replicated by all layers of cuticle (but allowing for some detachment of layers)	Morphology only visible in outer cuticle	No longer identifiable
Gonopore	Morphology faithfully replicated by all layers of cuticle (but allowing for some detachment of layers)	Morphology only visible in outer cuticle	No longer identifiable
Internal anatomy			
Epidermis	Thick white layer attached to procuticle and outer cuticle	Detached from procuticle and outer cuticle, thinned, but still forms distinct cavity	Too thin to identify and/or collapsed onto and indistinguishable from internal organs
Slime glands	As at death	Disaggregated, loss of endpieces	No longer identifiable, part of white mass
Gut	As at death	Soapy and easily disaggregated	No longer identifiable, part of white mass
Gonads	As at death	Indistinct outline, disaggregated	No longer identifiable, part of white mass
Nerve cords	Clear paired structures	Visible but indistinct	No longer visible
Body wall musculature	As at death	Visible but indistinct	No longer visible
