# Supplementary material for: Decay of velvet worms (Onychophora), and bias in the fossil record of lobopodians
Source: BMC Evol Biol. 2014 Nov 29;14:222. doi: 10.1186/s12862-014-0222-z (PMC4266977; doi:10.1186/s12862-014-0222-z)
Supplement: Additional file 9: — Table of phylogenetic distribution of characters among panarthropods and cycloneuralians. [file 12862_2014_222_MOESM9_ESM.pdf]

Additional file 9: Phylogenetic distribution of characters among panarthropods and cycloneuralians.

	Character in Onychophora	Comparison with Arthropoda	Comparison with Tardigrada	Comparison with Cycloneuralia
Overall morphology				
Integrity of cuticle	Decay resistant integument or cuticle	Homologue	Homologue	Homologue
Anterior	Differentiated region in front of anterior-most walking legs	Nature of the homology unclear	Nature of the homology unclear	-
Limbs	Lobose walking limbs	Homologue – arthropodized limbs derived	Homologue	-
Posterior	Differentiated region behind of posterior-most limbs	Homologue	Absent in modern forms, possible plesiomorphy	-
Head structures				
Antennae	Autapomorphy, possibly homologous to arthropod labrum	Analogous structure	-	-
Slime papillae	Autapomorphy	-	-	-
Jaws	Autapomorphy	-	-	-
Mouth (with tongue)	Ventral mouth	Analogous ventral mouth	Sub-terminal mouth	Analogous terminal mouth
Eyes	Ocellus-like eyes	Likely homologous with the median ocelli, but not compound eyes [17]	Homology disputed – eye spots	-
Trunk structures				
Dermal papillae	Autapomorphy or possible plesiomorphy (homologous to “tubercles” of e.g. *Aysheaia*)	-	-	-
Pigment	Autapomorphy	Analogous pigments not housed in granules	Analogous pigments not housed in granules	Analogous pigments not housed in granules

Trunk annuli (dermal plicae)	Autapomorphy	-	-	-
Limbs				
Transverse leg rings	Autapomorphy, possible plesiomorphy (cf. annulations of limbs of e.g. *Onychodictyon ferox* and *Aysheaia pedunculata*)	-	-	-
Dermal papillae on limbs	Autapomorphy, possible plesiomorphy (cf. limbs of *Orstenotubulus evamuellerae*)	-	-	-
Foot claws	Two claws per limb	-	Multiple claws	-
Feet	Autapomorphy	-	-	-
Posterior body region				
Anus	Present	Homologue	Homologue	Homologue
Gonopore	Present	Homologue	Homologue	Homologue
Internal anatomy				
Epidermis	Present	Homologue	Homologue	Homologue
Slime glands	Autapomorphy	-	-	-
Gut	Symplesiomorphy	Homologue	Homologue	Homologue
Gonads	Symplesiomorphy	Homologue	Homologue	Homologue
Nerve cords	Ventral nerve cords	Ventral chain of ganglia linked by somata-free connectives	Ventral chain of ganglia linked by somata-free connectives	Analogous nerve cords
Body wall musculature	Symplesiomorphy	Homologue	Homologue	Homologue
