# Supplementary material for: Decay of velvet worms (Onychophora), and bias in the fossil record of lobopodians
Source: BMC Evol Biol. 2014 Nov 29;14:222. doi: 10.1186/s12862-014-0222-z (PMC4266977; doi:10.1186/s12862-014-0222-z)
Supplement: Additional file 10: — Additional background, results and details of the methodology. [file 12862_2014_222_MOESM10_ESM.pdf]

Additional Information: Decay of velvet worms (Onychophora), and bias in the fossil record of lobopodians

Duncan J. E. Murdock[1], Sarah E, Gabbott[1], Georg Mayer[2] and Mark A. Purnell[1]*

[1] Department of Geology, University of Leicester, University Road, Leicester LE1 7RH, UK

[2] Animal Evolution and Development, Institute of Biology, University of Leipzig, Talstraße 33, D-04103 Leipzig, Germany

*Author for correspondence (mark.purnell@le.ac.uk)

Additional background

Due (in part) to the paucity of informative characters, the topology of the lobopodian tree is highly unstable. The addition of new taxa often results in significant perturbations (cf. Ref 1,2) and more thorough phylogenetic analyses frequently recover a widespread polytomy [1, 2]. Constraining which characters can be reliably applied to decayed fossilized remains is crucial for testing competing phylogenetic hypotheses, and resolve some outstanding questions in lobopodian evolution. The onychophoran affinity of Cambrian lobopodians was first proposed for the Burgess Shale taxon *Aysheaia* [3], which shares a range of supposed synapomorphies with onychophorans that distinguish it from other lobopodians, such as homonomous trunk annulation, lack of sclerotized plates and lack of differentiated tagmata. In a range of analyses the precise affinity of *Aysheaia* is highly variable, yet it is consistently resolved at or near the most basal positions (i.e. occupying the deepest branch in the stem-groups to the Arthropoda [4], Onychophora [5, 6], Lobopodia [7, 8] or Panarthropoda [9, 10]. These basal positions in trees imply that the suite of characters shared by onychophorans and *Aysheaia* are homoplasies, but the possibility that this phylogenetic position results from stemward slippage is untested. Lobopodian evolution also has

wider implications for the interpretation of the Palaeozoic biosphere and the colonization of terrestrial environments. Extant onychophorans are exclusively terrestrial, but their ancestors were marine; when did the transition occur? Understanding the potential of the fossil record to preserve the anatomical characters of crown-onychophorans has a direct bearing on this question.

Additional methods details

Euperipatoides rowelli were cultured in the laboratory from a stock collected in the Tallaganda State Forest (New South Wales, Australia; 35°26'S, 149°33'E, 954 m) in October 2009. *Principapillatus hitoyensis* were cultured in the laboratory from a stock collected in the Reserva Biológica Hitoy Cerere (Province of Limón, Costa Rica; 09°40'N, 83°02'W, 300 m) in October 2005. Specimens were transported live from Leipzig to Leicester in ventilated, screw-top, plastic jars containing peat and moist tissues. Individuals were kept alive in these vessels for between 2 days and 12 weeks with tissues replaced every 5 days. Experimental animals were fed with incapacitated third-molt silent crickets every 2–3 weeks, 24 hours after which their peat was replaced.

All experimental animals were euthanized using nitrogen asphyxia; sealed in a desiccator with a one-way valve, compressed oxygen-free nitrogen was slowly let in to replace the air. Pilot experiments indicated the largest individuals were torpid after 5 minutes of exposure, and dead after 20 minutes. The effect of temperature change due to decompression was reduced by keeping the animals in high-sided vessels and allowing the gas to be let in at the base of the desiccator.

The decay experiment methodology generally followed that of Sansom *et al.* [11, 12] with modifications to account for the terrestrial life habit of velvet worms. Onychophoran cuticle is water repellent, and in order to entirely wet the surface and avoid the inclusion of trapped air bubbles the experimental animals were briefly dipped in a 70% ethanol bath after euthanasia, then temporarily stored in a bath of buffer solution to remove residual ethanol. Specimens were decayed in individual 58 x 38 x 22 mm polystyrene boxes with push fit lids, filled with a physiological

buffer solution based on the composition of onychophoran hemolymph (= Robson's saline) [13], with the exception of glucose. This isotonic solution (~7.3 ‰) was used to limit the effect of osmosis during the experiment (see below). In order to minimize the number of experimental variables, no bacterial inoculae or antifungal agents were added, nor were any attempts made to disrupt the endogenous bacteria of the specimen. Experimental boxes were sealed with silicone grease M494 (ACC Silicones Ltd) to reduce gas exchange with the outside.

Specimens were photographed, then the experimental boxes were stored in temperature controlled incubators at 25°C (±1°C). Specimens were destructively sampled over 220 days, three per interval (*E. rowelli*), or one per interval (*P. hitoyensis*, due to limited specimen availability). Sampling intervals followed a logarithmic model, with high initial sampling frequency to capture early rapid decay. At each sampling interval specimens were photographed prior to and after dissection, and the condition of external and internal anatomical features logged and described. An expanded aluminium mesh floor (pores less than 2 x 2 mm) in each box aided in removal of specimens. For dissection, specimens were pinned to dental wax with entomological pins under distilled water. A subset of dissected samples was prepared for scanning electron microscopy by fixing with a 2.5% phosphate-buffered glutaraldehyde solution overnight at 4°C, dehydrating through a graded series of ethanol, and critical point drying, before being sputter-coated with gold. Imaging was carried out on a Hitachi S-3600N Environmental Scanning Electron Microscope.

Measurements of the length and width of the trunk, and length and width of the limbs (at the seventh limb pair) at day 0 and in each subsequent sample, were taken from scaled photographs using ImageJ v10.2 (National Institutes of Health, USA). For decayed specimens, separate measurements were made of the outer surface of the cuticle and the outer surface of the epidermis visible through the cuticle. Changes in overall size, shape and proportions through decay could then be assessed (figure 2).

Internal and external anatomy of onychophorans (figure 1) was documented and morphological characters were categorized by position in the organism: anterior, trunk, limbs,

posterior, and internal organs. For morphological decay, each character for each sample for each interval was scored according to three defined states: pristine (same condition as at death), decaying (morphology altered from that of condition at death) or lost (no longer observable or recognizable) (Additional files 3–5). To consistently apply these states to a range of tissue types, and to attribute equal weight to characters that are contingent upon others, individual definitions for each character state were required (see supplementary table S1).

For statistical analysis, characters were arranged according to the sequence in which they decay and ranked from the most decay-prone to the most decay-resistant (figure 2; additional file 4). The decay rank and synapomorphic rank of characters were then compared using Spearman's rank correlation, with synapomorphic rank calculated for each of four alternative hypotheses of panarthropod relationships. Where the homology of characters is contested, we conducted separate analyses treating them either as homologies or homoplasies. This yielded 8 correlation tests. The phylogenetic relationships between the panarthropod subgroups and the placement of lobopodian taxa on their stems is highly unstable, and a number of competing hypotheses exist [1, 2, 8, 10]. Considering only the relative relationships of arthropods, onychophorans, tardigrades and cycloneuralians, four alternative hypotheses are generally proposed (figure 5). These alternatives are considered here, and the characters observed in the decay experiments mapped onto each topology. Where there are ambiguous character distributions two alternatives are considered, one favoring homology of characters the other favoring convergent evolution (homoplasy). Consequently, eight phylogenetic hypotheses were compared to the decay ranks drawn from the experimental results. Two suites of characters can be regarded as phylogenetically uninformative for establishing relationships between stem and crown taxa: onychophoran autapomorphies and symplesiomorphies of the Ecdysozoa (additional file 9). Either may be biasing the analysis, so rank comparisons were also performed with each of these sets of characters removed. For decay data and phylogenetic hypotheses, characters with equal, or tied ranks were assigned scores by calculating mean values of their ordinal ranks (e.g. four characters joint third receive a score of 4.5).

The decay trajectory of characters was assessed by recording the time at which they reached specific thresholds of decay (figure 3). Where log transformation of sampling data were required for analysis, day zero was assigned a value of 0.04 (= 1 hour)

Accessory experiments were carried out to test for osmotic effects on post-mortem anatomical change in onychophorans, to test the effect of molt stage on post-mortem cuticle expansion, and to test for variation in decay between taxa. To test for osmotic effects we carried out a series of experiments with specimens of *E. rowelli* immersed in Robson's saline prepared to half (~3.7 ‰) and double (~14.6 ‰) concentration. To test the effect of moult stage on cuticle expansion, we decayed six individuals of *E. rowelli* under the same conditions as the main experiments, except that half were not fed for 2 weeks, and half were fed 48 hours before euthanasia (cultured specimens of *E. rowelli* are known to molt within 48 hours of feeding). The two groups were monitored for 20 days. To test for interspecific variation in decay, a small number (n=5) of individuals of the peripatid *P. hitoyensis*, was treated as in the main experiment.

Additional results

Decay can be characterized as a series of stages (figure 1, table 1). The first signs of decay are the breakdown of the procuticle and separation of the outer cuticle and the epidermis. This is seen through the bloating of the outer cuticle, shrinkage of the epidermis onto the internal organs (by up to 25%) and loss of pigmentation. Loss of strong pigmentation occurs within hours of death. The ventral surface becomes pink and ultimately white, whilst the blue pigment fades to pale blue, then, after several days, green. Initially the distribution of blue pigment is consistent across the dorsum, and is contained within granules, which are in place, but easily disturbed during dissection (figure 1). From day 6 onwards the pigment takes on a cracked and patchy appearance, although pigment is still contained within distinct granules. From around day 75 individual granules are no longer identifiable, and the dark-green coloration of the carcass can be attributed to a 'sludge' derived from the breakdown of pigment granules. Initially, annuli (=dermal plicae) are also visible on the

inner cuticle (i.e. the outer surface of the epidermis), marked by rows of pigment. However, with the further separation of the layers of the cuticle, and the breakdown of the pigment granules, the annuli are no longer visible on the inner cuticle after day 12, and there is no indication of either annulation or segmentation on the internal anatomy (figure 1).

The internal anatomy initially loses fine details (such as slime gland endpieces), then distinct boundaries between individual organs become difficult to discern. The thinning and collapse of the epidermis makes identification of muscles impossible by the end of decay stage 2. By day 12, the epidermis and internal organs have degraded into a white soapy mass, surrounded by fluid encapsulated by a thin outer cuticle. At day 109 the entire outer cuticle is filled with a white soapy mass and none of internal anatomy is distinguishable. This is followed by further swelling and ultimately rupturing. The rupture generally occurs where the carcass is in contact with the aluminium mesh, and often causes corrosion of the mesh. This is likely due to extreme fluctuations in local pH (note, no significant change in bulk pH of the water was observed) causing the loss of the thin film of aluminium oxide, which would otherwise prevent the corrosion of aluminium in water. On some occasions this corrosion occurred through thinning but no rupture of the cuticle, but was not observed before day 109 in any specimen.

The architecture of the outer cuticle is intact until at least decay stage 2, with sensilla on the antennae and sensory bristles, scales and other fine details still present (additional file 2a). The inner surface of the integument is initially smooth (additional file 2b), but early evidence of the breakdown of the procuticle is discernable from as early as day two, with separate layers visible (additional file 2c). By decay stage 4 the inner surface of the integument is no longer smooth, exposing the of circular and diagonal muscles fibers from beneath the epidermis (additional file 2d), and although thinned, the fine structure of the outer cuticle remains intact (additional file 2e). During decay stage 5, this is no longer the case; dermal papillae are still visible, but they have lost ornament and no fine-scale structure remains; the inner surface of the integument is rubbly in appearance (additional file 2f). By the end of decay stage 5, fixed and dried samples are difficult to

produce due to tissue degradation. Some sensory bristles remain, but the original textural character of the outer cuticle is lost, largely reduced to a rough reticulate pattern. Where observed, neither jaws (additional file 2g) nor claws (additional file 2h) show signs of decay in any samples.

From the data gathered in these experiments it is possible to produce a spectrum of relative decay resistance, which can be used to inform the interpretation of lobopodian fossils:

1) *Internal organs and tissues*. The majority of the internal anatomy (including muscles, slime glands, gonads and nervous system) is extremely prone to decay, lost early in these experiments. The loss of body musculature is associated with the thinning of the epidermis, and is no longer visible after the epidermis becomes totally transparent.

2) *Gut*. The gut as a distinct organ is short-lived, rapidly degrading into a 'soapy' substance indistinguishable from the rest of the internal anatomy.

3) *Epidermis*. The rapid breakdown of the epidermis and subsequent distortion of the overall morphology is likely to be a significant factor in the preservation of many lobopodian fossils.

4) *Pigment*. The pigment granules of onychophorans are relatively decay-prone, first losing their distinct color, then breaking up into smaller particles, taking on a cracked and patchy appearance. At this point the position of pigment within the organism no longer reflects the position *in vivo,* and the dark green coloration of the carcass can be attributed to a 'sludge' derived from the breakdown of pigment granules.

5) *Mouth and Eyes*. The relative timing of loss of these two important features is difficult to separate: the point at which they decay beyond recognition is nearly simultaneous. Both characters have significant implications for the affinity of lobopodian taxa [7, 10, 14]. In later stages of decay, the outline of the mouth is so distorted that it is only identifiable as the mouth due to the presence of jaws.

6) *Cuticle*. The outer cuticle, as has been demonstrated for polychaetes and crustaceans [15, 16], is relatively decay-resistant. Its rupture and break-up is a significant contributor to the

loss of many informative characters, and the timing of their loss is contingent upon the integrity of the cuticle. These characters can be divided into gross anatomy (e.g. morphology of the antennae, slime papillae, limb shape, trunk shape, posterior body extension, and presence of anus/gonopore) and the ornament and structure of the cuticle itself (e.g. annulations and dermal papillae of the trunk and limbs, fine structure of the surface of the feet on limbs).

7) *Sclerotized jaws and claws*. The jaws and claws of *E. rowelli* show no decay on the timescale of our experiment; this compares to results of previous experiments on polychaete worms [15], where sclerotized jaws and setae remain when all other anatomy is lost to decay.

8) *Mineralized plates, sclerites or spines*. Although absent in modern onychophorans, mineralized tissues, such as the sclerites of *Microdictyon*, under most circumstances would have been the most decay-resistant parts of extinct lobopodians. Their higher abundance in the fossil record relative to preserved soft tissues is consistent with this.

Experimental analyses of soft-tissue decay have been carried out previously on the polychaete annelid *Nereis* [15] and the crustacean arthropods *Crangon* and *Palaemon* [16]. With the addition of the onychophoran data here a number of common patterns can be identified. Initially the cuticle is rigid, body cross-section retains original three-dimensionality and pigmentation is intact. As decay proceeds the cuticle loses its rigidity and, due to osmotic effects, either collapses (*Nereis*) or detaches from the underlying epidermis (*Crangon*, *Palaemon* and *Euperipatoides rowelli*). Pigmentation begins to degrade and disperse. In more advanced stages of decay the internal anatomy liquefies, leaving a cuticle sac, which ultimately ruptures, empties and disarticulates. Finally only fragments of cuticle and the most decay-resistant structures remain, polychaete jaws and setae, crustacean eye pigments and, by inference, onychophoran jaws and claws.

References

1. Legg DA, Ma X, Wolfe JM, Ortega-Hernández J, Edgecombe GD, Sutton MD: **Lobopodian phylogeny reanalysed**. *Nature* 2011, **476**:E1–E1.

2. Mounce RCP, Wills MA: **Phylogenetic position of *Diania* challenged**. *Nature* 2011, **476**:E1–E1.

3. Hutchinson GE: **Restudy of some Burgess Shale fossils**. *Proc United States Natl Museum* 1930, **78**:1–24.

4. Liu JN, Shu D, Han J, Zhang ZF, Zhang XL: **Morpho-anatomy of the lobopod Magadictyon cf. haikouensis from the Early Cambrian Chengjiang Lagerstatte, South China**. *Acta Zool* 2007, **88**:279–288.

5. Hou X, Ramsköld L, Bergström J: **Composition and preservation of the Chengjiang fauna - a Lower Cambrian soft-bodied biota**. *Zool Scr* 1991, **20**:395–411.

6. Poinar G: **Fossil onychophorans from Dominican and Baltic amber: *Tertiapatus dominicanus* n.g., n.sp (Tertiapatidae n.fam.) and *Succinipatopsis balticus* n.g., n.sp (Succinipatopsidae n.fam.) with a proposed classification of the subphylum Onychophora**. *Invertebr Biol* 2000, **119**:104–109.

7. Liu JN, Shu D, Han J, Zhang ZF: **A rare lobopod with wellpreserved eyes from Chengjiang Lagerstatte and its implications for origin of arthropods**. *Chinese Sci Bull* 2004, **49**:1063–1071.

8. Liu J, Steiner M, Dunlop JA, Keupp H, Shu D, Ou Q, Han J, Zhang Z, Zhang X: **An armoured Cambrian lobopodian from China with arthropod-like appendages**. *Nature* 2011, **470**:526–530.

9. Daley AC, Budd GE, Caron J-B, Edgecombe GD, Collins D: **The Burgess Shale Anomalocaridid *Hurdia* and Its Significance for Early Euarthropod Evolution**. *Science* 2009, **323**:1597–1600.

10. Ma X, Hou X, Bergström J: **Morphology of Luolishania longicruris (Lower Cambrian, Chengjiang Lagerstatte, SW China) and the phylogenetic relationships within lobopodians**. *Arthropod Struct Dev* 2009, **38**:271–291.

11. Sansom RS, Gabbott SE, Purnell MA: **Decay of vertebrate characters in hagfish and lamprey (Cyclostomata) and the implications for the vertebrate fossil record**. *Proc R Soc London Ser B Biol Sci* 2011, **278**:1150–1157.

12. Sansom RS, Gabbott SE, Purnell MA: **Non-random decay of chordate characters causes bias in fossil interpretation**. *Nature* 2010, **463**:797–800.

13. Robson EA: **Composition of the Blood in Onychophora**. *Nature* 1966, **209**:553.

14. Ou Q, Shu D, Mayer G: **Cambrian lobopodians and extant onychophorans provide new insights into early cephalization in Panarthropoda**. *Nat Commun* 2012, **3**:1261.

15. Briggs DEG, Kear AJ: **Decay and preservation of polychaetes - taphonomic thresholds in soft-bodied organisms**. *Paleobiology* 1993, **19**:107–135.

16. Briggs DEG, Kear AJ: **Decay and mineralization of shrimps**. *Palaios* 1994, **9**:431–456.

17. Mayer G: **Structure and development of onychophoran eyes: What is the ancestral visual organ in arthropods?** *Arthropod Struct Dev* 2006, **35**:231–245.
